# Harnessing the Antibacterial, Anti-Diabetic and Anti-Carcinogenic Properties of *Ocimum sanctum* Linn (Tulsi)

**DOI:** 10.3390/plants13243516

**Published:** 2024-12-16

**Authors:** Rakesh Arya, Hossain Md. Faruquee, Hemlata Shakya, Sheikh Atikur Rahman, Most Morium Begum, Sudhangshu Kumar Biswas, Md. Aminul Islam Apu, Md. Azizul Islam, Md. Mominul Islam Sheikh, Jong-Joo Kim

**Affiliations:** 1 Department of Biotechnology, Yeungnam University, Gyeongsan 38541, Republic of Korea; rakesharya101@yu.ac.kr; 2 Department of Biotechnology and Genetic Engineering, Faculty of Biological Science, Islamic University, Kushtia 7003, Bangladesh; skatikurrahman347@gmail.com (S.A.R.); skbiswas@btge.iu.ac.bd (S.K.B.); azizul@btge.iu.ac.bd (M.A.I.); 3 Department of Biomedical Engineering, Shri G. S. Institute of Technology and Science, Indore 452003, India; hemlata.shakya19@gmail.com; 4 Department of Agriculture, Rabindra Maitree University, Kushtia 7000, Bangladesh; moriumru@gmail.com; 5 Department of Nutrition and Hospitality Management, The University of Mississippi, Oxford, MS 38677, USA; mapu@go.olemiss.edu; 6 Nano Project Group, Research & Development Institute, Moorim P&P Co., Ltd., Ulju-gun, Ulsan 45011, Republic of Korea; momin.gnu@gmail.com

**Keywords:** *Ocimum sanctum* Linn, eugenol, anti-inflammatory, antioxidants, radioprotective

## Abstract

*Ocimum sanctum* Linn (*O. sanctum* L.), commonly known as Holy Basil or Tulsi, is a fragrant herbaceous plant belonging to the Lamiaceae family. This plant is widely cultivated and found in north-central parts of India, several Arab countries, West Africa and tropical regions of the Eastern World. Tulsi is known to be an adaptogen, aiding the body in adapting to stress by harmonizing various bodily systems. Revered in Ayurveda as the “Elixir of Life”, Tulsi is believed to enhance lifespan and foster longevity. Eugenol, the active ingredient present in Tulsi, is a l-hydroxy-2-methoxy-4-allylbenzene compound with diverse therapeutic applications. As concerns over the adverse effects of conventional antibacterial agents continue to grow, alternative therapies have gained prominence. Essential oils (EOs) containing antioxidants have a long history of utilization in traditional medicine and have gained increasing popularity over time. Numerous in vitro, in vivo and clinical studies have provided compelling evidence supporting the safety and efficacy of antioxidant EOs derived from medicinal plants for promoting health. This comprehensive review aims to highlight the scientific knowledge regarding the therapeutic properties of *O. sanctum*, focusing on its antibacterial, anti-diabetic, anti-carcinogenic, radioprotective, immunomodulatory, anti-inflammatory, cardioprotective, neurogenesis, anti-depressant and other beneficial characteristics. Also, the extracts of *O. sanctum* L. have the ability to reduce chronic inflammation linked to neurological disorders such as Parkinson’s and Alzheimer’s disease. The information presented in this review shed light on the multifaceted potential of Tulsi and its derivatives in maintaining and promoting health. This knowledge may pave the way for the development of novel therapeutic interventions and natural remedies that harness the immense therapeutic potential of Tulsi in combating various health conditions, while also providing valuable insights for further research and exploration in this field.

## 1. Introduction

**Figure 1:**
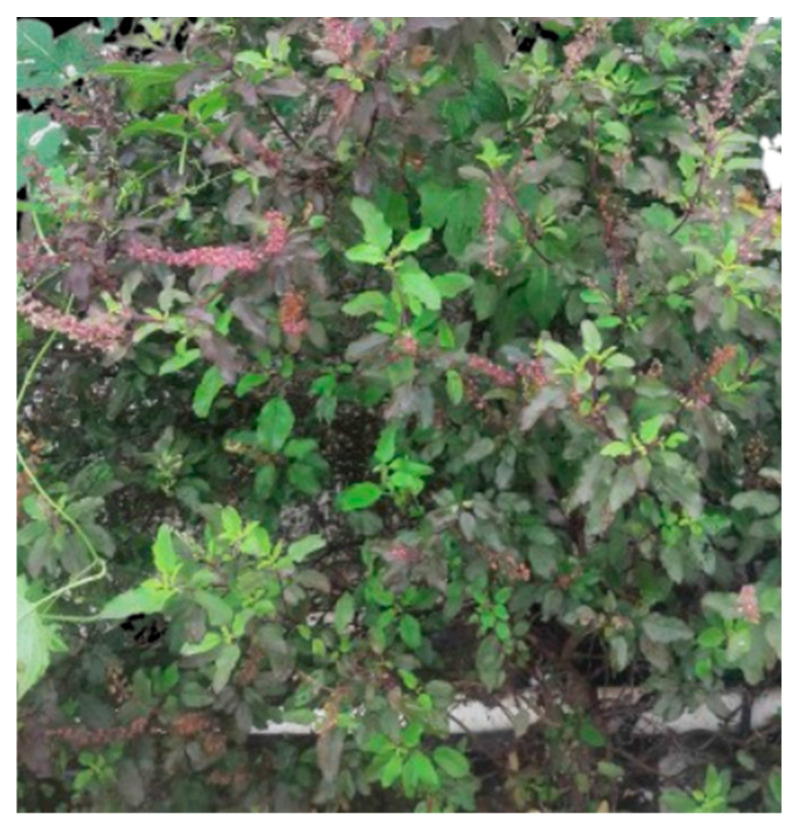
*O. sanctum* L. (Tulsi) plant.

Natural compounds’ ability to maintain and improve human health has piqued the curiosity of researchers. Nature has long provided an extensive variety of treatments for many human diseases, with traditional medicine acting as the major source of basic medical requirements for more than 80% of the world’s population [1]. For thousands of years, traditional practitioners have employed medicinal plants to cure a wide range of diseases [2]. According to a survey conducted by the World Health Organization (WHO), traditional medicine practitioners treat a significant number of patients in countries such as India (80%), Burma (85%) and Bangladesh (90%) [3]. Holy Basil, commonly known as Tulsi, has a long history of traditional usage across numerous medicinal systems. Much research has looked at the immunomodulatory activities of *O. sanctum* L. and its bioactive components [4,5]. In Ayurvedic medicine, Holy Basil is known as the “Queen of Herbs” and the “Elixir of Life” [6]. This distinctive subshrub grows erect with abundant branching and a height of 30 to 60 cm. Its stems are covered in fine hairs and its aromatic green or purple leaves develop in pairs. The flowers of *O. sanctum* L. produce elongated clusters in compact whorls and are a distinctive shade of purple. The ovate leaves are connected to the stem by their petioles (Figure 1).

Holy Basil’s therapeutic potential is astounding. This remarkable plant’s (different parts) immunotherapeutic mechanisms are elucidated in detail in an exhaustive review that reported the phytochemicals found in *O. sanctum* L., such as eugenol, rosmarinic acid and ursolic acid, which exhibit immunomodulatory activities that can influence immune cell functions and boost immune responses [7,8]. Traditional medical literature such as the Indian Materia Medica mentioned Tulsi leaf preparations for the treatment of pyrexia, rheumatism and bronchitis [9]. The immunomodulatory properties of *O. sanctum* L. extracts and compounds have been studied extensively. For example, administering *O. sanctum* L. leaf extract stimulated the synthesis of numerous immune cells, such as lymphocytes and macrophages, resulting in an improved immunological response and protection against infections [9,10]. *O. sanctum* L. offers immunotherapeutic promise for a variety of diseases. Recent research highlighted its impact in lowering oxidative stress and inflammation, which improves immune system performance and protects against chronic diseases [11]. Furthermore, studies indicate that *O. sanctum* L. can improve the efficiency of cancer immunotherapies by boosting the activation and proliferation of immune cells responsible for tumor eradication [12]. A number of studies revealed that the plant has been used historically to treat epilepsy, asthma or dyspnea, hiccups, congestion, skin and hematological diseases, parasite infections, neuralgia, migraines, wounds, inflammation, and dental issues (Table 1) [13,14,15]. Its medicinal properties make it a valuable resource for addressing a vast array of health issues across cultures. The leaf juice is reportedly used in traditional medicine to treat earaches, while the root and stems are employed to treat serpent and insect bites [16,17]. In Ayurvedic treatments for malaria, poisoning, gastrointestinal issues, colds and gastric problems, *O. sanctum* L. extracts have been utilized [18]. Tulsi, with its numerous secondary metabolites, essential oils and therapeutic properties, has influenced numerous facets of human existence, including dietary customs [19]. Bioactive compounds such as camphor, eucalyptol, eugenol, alpha-bisabolene, beta-bisabolene and beta-caryophyllene are abundant in Tulsi essential oil [20,21]. It is believed that these compounds are responsible for the antimicrobial properties of leaf extracts [21]. It has been demonstrated that Tulsi leaves, which contain high concentrations of eugenol, have anticancer properties. Eugenol exerts its antitumor effects via a variety of mechanisms in a variety of malignancies [22]. For health benefits, *O. sanctum* L. fresh leaves, dried powder and herbal tea are ingested [23]. Additionally, dried Tulsi leaves have been used for centuries to repel insects from stored cereals [24]. *O. sanctum* L. has significant immunotherapeutic potential, making it a valuable natural resource for maintaining good health. Numerous studies have demonstrated its immunomodulatory effects, indicating its capacity to enhance immune response, modulate inflammation and combat oxidative stress. To elucidate the precise mechanisms of action and explore the full potential of *O. sanctum* L. as an immunotherapeutic agent, additional research and clinical trials are required.

## 2. Phytochemical Constituents

**Figure 2:**
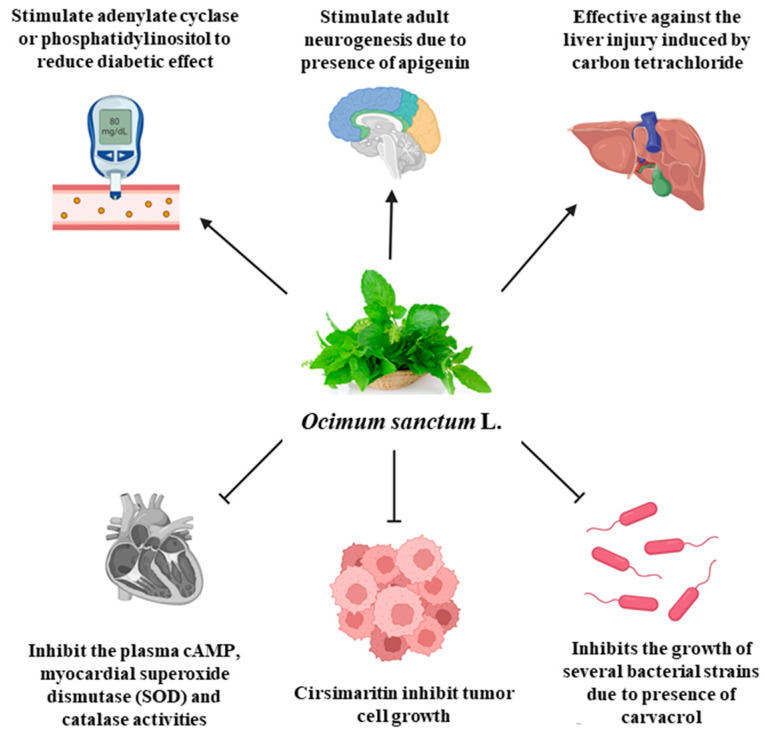
The schematic diagram illustrates the different medicinal properties of *O. sanctum* L. (Tulsi). Each of these activities highlights the diverse therapeutic potential of Tulsi, making it a valuable nutraceutical agent in promoting health and managing different diseases. (Figure created with biorender.com).

*O. sanctum* L. has very sophisticated chemical characteristics that contain a wide range of nutrients and other physiologically active substances [29]. Bioactive chemicals are abundant throughout the entire plant parts. The chemical composition of *O. sanctum* L. (Holy Basil) is quite complex, and the levels of various compounds can be influenced by factors such as cultivation, harvesting, processing and storage conditions [30]. According to reports, the leaves of *O. sanctum* L. contain roughly 0.7% volatile oil, which mostly comprises around 71% eugenol and 20% methyl eugenol [31,32,33]. Carvacrol and the sesquiterpine hydrocarbon caryophyllene are also present in the oil [31]. Its fresh leaves and stem include phenolic substances such as cirsilineol, circimaritin, isothymusin, apigenin and rosmarinic acid, as well as significant amounts of eugenol. Orientin, vicenin, ursolic acid, luteolin, apigenin-7-O-glucuronide, luteolin-7-O-glucuronide and molludistin are also found in the leaves [34] (Figure 2) (Table 2).

## 3. Nutraceutical Value

Dietary minerals have a crucial role in both food and nutraceutical industries. The herb *O. sanctum* L. has long been utilized as a flavoring agent in food and as a home remedy for various health conditions. This plant is known for its abundant content of nutraceutical properties [29,38,39]. *O. sanctum* L. contains a variety of essential nutrients and phytonutrients, including vitamin A, beta-carotene, vitamin C, insoluble oxalates, chlorophyll, fat, protein, minerals, carbohydrates and other beneficial compounds. In a 100 g serving of Holy Basil leaves, one can find 83 µg of vitamin C, 3.15% of calcium, 2.5 µg of carotene, 2.9 µg of chromium, 0.4 µg of copper, 0.34% of phosphorus, 0.54 µg of vanadium, 0.15 µg of zinc, 0.73 µg of nickel and 2.32 µg of iron [29]. The determined value of total carotenoid content is 19.77 g in 100 g, the total flavonoid content is 1.87 g in 100 g and the total phenolic content is 2.09 g in 100 g of dry weights in *O. sanctum* L. leaves [40]. *O. sanctum* L. leaves are a viable option for dietary supplementation and serve as a natural antioxidant due to their significant content of ascorbic acid 8.21 mg in 100 g, thiamine 0.3 mg in 100 g and riboflavin 0.06 mg in 100g. Moreover, they offer an economical alternative source of vitamins, making them beneficial for overall health and well-being [19]. Recent research continues to unveil the diverse therapeutic potential of Tulsi, consolidating its position as a valuable nutraceutical agent in contemporary health care practices (Table 3).

## 4. Pharmacological Activity

### 4.1. Effects on Gene Transcription

Recent developments in the field of genetic research have led to the discovery of a number of important genes, such as LDLR (Low-Density Lipoprotein Receptor), Liver X receptor alpha, Peroxisome proliferator-activated receptors (PPARs) and CD-36, which all play an important role in the development of atherosclerosis [48]. These genes contribute to the establishment and evolution of atherosclerotic lesions by regulating critical activities within the arterial wall, such as the manufacture of cytokines, the metabolism of lipids and cellular activity [49]. In this setting, innovative therapeutic treatments focusing on the modification of gene expression have garnered a lot of attention. The natural polyphenol extract found in Tulsi are found to block the expression of these genes during the transcription process [50].

### 4.2. Anti-Toxic Effect

Studies have demonstrated the ability of Holy Basil to counteract the toxic effects of heavy metals, environmental pollutants and other toxic substances [51]. Cadmium is a heavy metal that is extremely hazardous, even at low concentrations. It can cause both chronic and acute health problems and it is known to have a negative impact on the liver in addition to other organs [52]. Cadmium can enter the body through inhalation or ingestion, at which point it is able to interfere with sulfhydryl-containing metabolic enzyme systems in the liver and disrupt mitochondrial oxidative phosphorylation [53]. This can lead to hepatic congestion, as well as ischemia and hypoxia. The ability of the hydroalcoholic extract of *O. sanctum* L. to scavenge free radicals and alter the antioxidant state led to tissue regeneration in the liver of rats that had been exposed to cadmium [54]. This ability may be related to the antioxidant activity of the extract. The extract showed a dose-dependent inhibition of the deleterious effects of cadmium on the antioxidant status of the liver, demonstrating its potential to counteract oxidative damage [55]. This confirmed the extract’s ability to protect against oxidative damage. Inflammation is a common response to toxic insults. Holy Basil exhibits anti-inflammatory properties, suppressing inflammation induced by toxins. It is reported that Swiss albino mice receiving oral *O. sanctum* L. extract were protected against HgCl_2_-induced toxicity [56].

### 4.3. Antioxidant Activity

Oxidative damage has the ability to affect DNA, as well as proteins, membrane lipids, and carbohydrates [57]. Multiple diseases like cancer, liver cirrhosis, diabetes, and atherosclerosis have been linked to oxidative damage [58]. The extract obtained from *O. sanctum* L., on the other hand, has an amazing ability to effectively neutralize these extremely reactive free radicals [59]. The presence of phenolic chemicals in the extract obtained from the fresh leaves and stems of *O. sanctum* L. is likely responsible for the outstanding antioxidant qualities that this extract possesses. These substances, such as cirsimaritin, cirsilineol, apigenin, isothymusin, rosmarinic acid, and considerable quantities of eugenol, play an important role in increasing the antioxidant activity of the extract. Other chemicals that contribute to this enhancement include rosmarinic acid [60].

### 4.4. Anticoagulant Activity

The anticoagulant action of *O. sanctum* L. fixed oil is particularly interesting due to its possible medicinal uses. The anticoagulant properties of *O. sanctum* L. fixed oil were compared to those of aspirin, a well-known anticoagulant drug. The intraperitoneal dose of 3 mL/kg of *O. sanctum* L. fixed oil resulted in an increase in blood coagulation time, which was attributed to its capacity to suppress platelet aggregation [61]. The similar outcome was seen when 100 mg/kg of aspirin was given. The findings suggest that *O. sanctum* L. fixed oil may have promise as a natural anticoagulant agent, and its therapeutic uses need further research [62].

### 4.5. Antifertility Agent

Ursolic acid, found in high concentrations in the leaves of *O sanctum* L., has the ability to inhibit estrogenic activity and acts as an antifertility drug [63]. It has been demonstrated to reduce spermatogenesis in males and to impair ovum implantation in females, which makes it a promising and safe antifertility treatment option. In addition, feeding tests conducted over extended periods of time on animal models demonstrated significant reductions in reproductive organ weights, sperm count and semen parameter values. However, it is notable that these alterations may be reversed, as shown by successful pregnancies in female rabbits after they stopped consuming *O. sanctum* L. leaves for a month [64]. This demonstrates that the nature of these changes can be reversed. It is necessary to conduct additional studies in order to investigate the mechanisms and therapeutic applications of *O. sanctum* L. and the ursolic acid that makes up its active component in the context of infertility therapies.

### 4.6. Activity Against Different Diseases

#### 4.6.1. Nephrolithiasis

Kidney stones, a common urological problem, can be extremely painful and necessitate surgical intervention to remove. The ability of a combination of honey and the juice of *O. sanctum* L. (Holy Basil) basil leaves to help kidney stones pass through the urinary tract was examined. In less than six months, the administration of honey and *O. sanctum* L. basil leaf juice can result in the effective transit of kidney stones. This natural cure provides a safe and effective alternative to conventional treatment approaches for the non-invasive management of kidney stones [65].

#### 4.6.2. Cardioprotective Effect

Psychological stress has emerged as a major contributor to cardiovascular risk, highlighting the need to investigate effective cardioprotective measures. Holy Basil (*O. sanctum* L.) was observed to have a prospective cardioprotective effect [66]. Psychological stress activates the sympathoadrenal and hypothalamus–pituitary–adrenal (HPA) axes, which results in the release of catecholamines, glucocorticoids and excitatory amino acids (EAAs). Long-term exposure to these substances can cause oxidative stress and adverse health effects. However, studies have demonstrated that Holy Basil inhibits the elevated plasma cAMP levels and the activities of cardiac superoxide dismutase (SOD) and catalase caused by chronic restraint stress (CRS) (Figure 2) [67]. These findings implied that Holy Basil may possess significant cardioprotective properties, suggesting that it could be used as a natural remedy to mitigate the negative cardiovascular effects of psychological stress. This herb’s primary component, eugenol, gives *O. sanctum* L. its remarkable antimalarial capabilities (Table 4) [68].

#### 4.6.3. Anticancer Effects

Cancer continues to be a significant threat to global health, necessitating the investigation of novel therapeutic approaches. Holy Basil’s potential anticancer properties have been studied [70]. Holy Basil has been shown in numerous preclinical and clinical studies to inhibit cancer cell growth, induce apoptosis or programmed cell death and modulate multiple molecular pathways implicated in cancer development and progression. Holy Basil extracts and their bioactive compounds activate apoptotic pathways, resulting in the selective elimination of cancer cells while sparing normal cells [70,71].

### 4.7. Antiviral Activity

Many important compounds of *O. sanctum* L. act as nontoxic antiviral agents that do not adversely affect the normal cellular mechanism [72]. Irrespective of the type of nucleic acid, viruses are sensitive to different constituents of *O. sanctum* L. extracts (Table 5).

### 4.8. Sexually Transmitted Diseases

Clinical isolates and strains from the WHO of *Neisseria gonorrhoeae* were inhibited by the purified fractions of the hexane extract of leaves of *O. sanctum* L. The active compound was characterized and determined to be eugenol, with a minimum inhibitory concentration (MIC) of 85–256 mg/L [74]. The activity is comparable to that of ciprofloxacin and penicillin [29].

### 4.9. Anticancer Activity

Lipids and oxygen-free radicals can combine to form hydroperoxides and peroxides, which can then negatively affect biological systems and cause cancer. Flavonoids can also stop free radicals from forming by chelating or complexing with the transition metal-free radical initiator. *O. sanctum* L. leaf extract inhibits chemical carcinogenesis through one or more mechanisms: Phase I and II enzymes are modulated; antioxidant activity is displayed and antiproliferative activity is displayed (Table 6) [75].

### 4.10. Liver Protective Behavior

Oral administration of a hydroethanolic extract obtained from the leaves of *O. sanctum* L. at a dosage of 200 mg/kg provided protective effects against paracetamol-induced liver injury in male Wistar albino rats. In experiments conducted on albino rats, it was observed that the whole plant or the leaves of *O. sanctum* L., when administered orally at a dosage of 3 g per 100 g body weight for a duration of 6 days, exhibited significant efficacy in protecting against liver damage induced by carbon tetrachloride (Figure 2). The assessment of hepatoprotective effectiveness was based on various parameters, including the ratio of blood proteins to albumin globulin, levels of alkaline phosphatase and transaminases, as well as the examination of liver histology [78].

### 4.11. Immunomodulatory Activity

Numerous studies have suggested that *O. sanctum* L. possesses immunomodulatory effects. The immunomodulatory effects of *O. sanctum* L. seed oil, which can affect both humoral and cell-mediated immune responses, are thought to occur through GABAergic pathways [79]. *O. sanctum* L. demonstrated increased antibody production in research. It might be caused by *O. sanctum* L. releasing mediators of tissue responses and hypersensitivity reactions in the target organs [80]. In addition, the treatment successfully boosted the production of red blood cells (RBCs), white blood cells (WBCs) and hemoglobin levels without causing any disruptions to the biochemical parameters [81].

### 4.12. Effect on Central Nervous System

Research has demonstrated that Holy Basil extracts can mitigate inflammation by regulating the expression of inflammatory mediators and signaling pathways, such as the nuclear factor kappa B (NF-κB) pathway. This modulation is instrumental in the reduction of chronic inflammation, which is frequently associated with neurodegenerative diseases and mood disorders, including depression [82]. Research has shown that Holy Basil extracts can strengthen the body’s antioxidant defenses, thereby safeguarding neuronal cells from apoptosis induced by oxidative stress. For example, research has demonstrated that Holy Basil can substantially reduce oxidative stress markers in a variety of models, including those that involve H_2_O_2_-induced neurotoxicity [83,84]. Recent clinical studies have investigated the impact of Holy Basil on neurogenesis and cognitive performance. A study administered 300 mg of an ethanolic extract daily for 30 days, revealing significant enhancements in cognitive metrics, including reaction time and error rates in tasks relative to a placebo group, as well as reductions in salivary cortisol levels, signifying improved cognitive performance and stress management [85]. Separate research with a standardized extract of 125 mg administered bi-daily for eight weeks demonstrated significant decreases in felt stress and enhancements in sleep quality, as indicated by reduced hair cortisol levels and increased ratings on sleep quality evaluations [86]. These results indicate that Holy Basil may possess potential neuroprotective and cognitive-enhancing properties in humans.

#### 4.12.1. Anti Stress

The constant state needed for successful adaptation must be maintained to respond to stress. *O. sanctum* L. leaf ethanolic extract was found to have anti-stress activity in the presence of both acute stress (AS) and chronic unpredictable stress (CUS) [82].

#### 4.12.2. Neuroprotective

*O. sanctum* L. has been demonstrated to change lipid profiles in the brain, which may contribute to its neuroprotective qualities during ischemia episodes [83].

#### 4.12.3. Anxiolytic

A study discovered that both aqueous and ethanolic extracts of *O. sanctum* L. significantly reduced anxiety levels in male albino rats exposed to restraint stress [84]. These effects were similar to those induced by diazepam, a well-known anxiolytic medicine, indicating that *O. sanctum* L. may function through similar neurochemical pathways, possibly including the regulation of serotonin and dopamine neurotransmitter systems [85].

#### 4.12.4. Anti Depression

Inflammation plays a pivotal role in the onset of depression, with increased concentrations of pro-inflammatory cytokines such as IL-6 and TNF-α associated with depressed symptoms. Holy Basil has demonstrated potential in this regard owing to its anti-inflammatory characteristics. Research suggests that Holy Basil can markedly diminish inflammatory indicators, perhaps mitigating neuroinflammation linked to depression. Moreover, studies have shown that Holy Basil extracts can reduce stress and anxiety, increase sleep quality, and promote general mental health. A randomized controlled trial lasting 8 weeks shown that Holy Basil supplementation resulted in reduced cortisol levels and enhanced subjective stress assessments [86]. Holy Basil antidepressant effects are due to its activity on the monoaminergic system, specifically the serotonin and dopamine pathways. In a study of mice using the Tail Suspension Test (TST), the ethanolic extract of *O. sanctum* L. at 150 mg/kg dramatically decreased immobility time, demonstrating considerable antidepressant efficacy. Mechanistic experiments demonstrated that pre-treatment with serotonin synthesis inhibitors (pCPA) and dopamine antagonists (Haloperidol and Sulpiride) reduced the extract’s potency. These findings imply that *O. sanctum* L. may increase serotonin levels and regulate dopamine receptor activation, suggesting its potential as a natural antidepressant for treating depression [87].

#### 4.12.5. Neurogenesis

Apigenin and related compounds found in Tulsi promote adult neurogenesis and also used for the treatment of neurological diseases, disorders and injuries, by promoting the development of new nerve cells in the adult brain (Figure 2) [88]. Apigenin increases the neurotrophic activities of BDNF (brain-derived neurotrophic factor) through direct binding, which may serve as a possible treatment for its curative efficiency in neurodegenerative diseases and depression [89]. Kalivarathan et al. suggested that apigenin could modulate brain insulin signaling during calorie excess by upregulating BDNF signaling through its ability to enhance GLP (Glucagon-like polypeptide)-1 that helps in insulin secretion [90]. Research indicates that apigenin provides neuroprotective effects in experimental models by modulating a number of signaling pathways implicated in oxidative stress, inflammation, and cell death. Several studies have highlighted the role of apigenin in neurodegenerative diseases (multiple sclerosis, Parkinson’s disease, Alzheimer’s disease), cancer, cardio-vascular diseases, cognitive and memory disorders, and toxicity related to trace metals and other chemicals [91].

### 4.13. Chemoprotective Activity

Orally and topically applied aqueous and ethanolic extracts of *O. sanctum* L. leaves were found to greatly delay the occurrence of papillomas and carcinomas. Furthermore, the survival rate was enhanced in individuals who re-received these extracts [92]. Hamster buccal pouch Carcinogenesis may be averted by utilizing an aqueous extract of *O. sanctum* L. leaves. The discovered mechanism for the extracts’ therapeutic benefits might be linked to an improved mixed function oxidase system (MFO), higher glutathione levels, and increased glutathione-S-transferase enzyme activity. These findings indicate that the extracts may have a role in detoxifying carcinogens, likely contributing to their capacity to postpone the formation of papillomas and carcinomas [79]. Additionally, the chemopreventive action of *O. sanctum* L. seed oil has been reported [93].

### 4.14. Anti-Tussive Activity

*O. sanctum* L. basic forms are commonly used as an expectorant and cough remedy, both alone and in combination with other herbs. To evaluate the possible antitussive (cough-suppressing) activity, guinea pigs exposed to an aerosol containing 7.5% *w*/*v* citric acid were administered aqueous and methanolic extracts of the plant [94]. In comparison to the methanolic extract (35.39% cough inhibition), the aqueous extract exhibited the strongest anti-tussive effect (72.5%). *O. sanctum* L. anti-tussive function is assumed to be a central action, possibly mediated by both the opioid and GABAergic systems [79].

### 4.15. Memory-Enhancing Activity

Preparations from *O. sanctum* L. are said to benefit patients with dementia and Alzheimer’s disease [79]. *O. tenuiflorum* (*O. sanctum* L.) ethanolic leaf extract increased step-down latency and inhibited brain acetylcholinesterase activity in mice with age- and scopolamine-induced amnesia, revealing the cholinergic foundation of *O. tenuiflorum*’s memory-enhancing effect [95].

### 4.16. Anti-Cataract Activity

In a 40-day trial of rats with galactose-induced cataracts and rabbits with naphthalene-induced cataracts, oral administration of *O. sanctum* L. resulted in a considerable delay in the onset and maturation of cataracts. Both models showed a 30% delay in galactose-induced cataracts in rats and a delay in naphthalene-induced cataracts in rabbits at a dosage of 1 g/kg naphthalene [96].

### 4.17. Anti-Diabetic Effect

Despite the use of several animal models to evaluate anti-diabetic efficacy, very few clinical studies have been performed. The use of *O. sanctum* L. in the treatment of diabetes mellitus has become increasingly pertinent [97]. The high fiber content of soluble plant fibers has demonstrated promise in the treatment of hyperglycemia and hyperlipidemia. The anti-diabetic activity of *O. sanctum* L. is thought to be mediated via the plant’s extract activating adenylate cyclase or phosphatidylinositol (Figure 2). Alternatively, it can have a direct influence on pancreatic cells by increasing calcium entry. These effects eventually result in the release of stored insulin, adding to the plant’s anti-diabetic qualities [79]. The leaves of the *O. sanctum* L. plant have considerable promise for enhancing insulin secretion and boosting pancreatic beta cell activity, which will help diabetic patients monitor their blood sugar levels. In diabetic mice induced by streptozotocin, an ethanol extract of *O. sanctum* L., derived from either the leaves or the entire plant, has shown potential to reduce blood glucose, urea and glycosylated hemoglobin levels. Furthermore, it has been observed to increase hemoglobin, glycogen and protein levels in these diabetic mice. Furthermore, the ethanol extract of *O. sanctum* L. has been found to elevate the levels of glucose and peptides and improve insulin tolerance [28]. *O. sanctum* L. possesses aldose reductase activity, which could assist in decreasing the effects of diabetes-related problems like cataract and retinopathy [98]. *O. sanctum* L. exhibits antiperoxidative properties and lowers cortisol and glucose levels in the blood, possibly regulating the development of corticosteroid-induced diabetes mellitus [99].

### 4.18. Radioprotective Activity

The radioprotective effect of OS was first documented in 1995. Orientin and vicenin, two isolated flavonoids from *O. sanctum* L. leaf extracts, outperformed artificial radioprotectants in terms of radioprotective effectiveness [100]. In the current study, the researchers investigated the potential radioprotective effects of *O. sanctum* L. on the salivary gland of rats exposed to radioiodine (^131^I) and evaluated the effectiveness of the plant against another well-known radioprotectant, amifostine. The study’s main finding is that both *O. sanctum* L. and amifostine have radioprotective effects on the salivary glands of rats exposed to therapeutic doses of radioiodine (^131^I) [101].

### 4.19. Activity That Lowers Lipids

Increased serum cholesterol can promote atherosclerosis by limiting blood supply to the organs, compromising organ function [102]. *O. sanctum* L. can dramatically increase high-density lipoprotein (HDL) cholesterol and total fecal sterol levels while significantly reducing triglycerides, total blood cholesterol, low-density lipoprotein (LDL) cholesterol and phospholipids. EO has been reported to dramatically diminish glutathione peroxidase (GPx), superoxide dismutase (SOD) and thiobarbituric acid reactive substances (TBARS) in cardiac tissue without altering catalase (CAT) [103].

### 4.20. Anti-Inflammatory Activity

*O. sanctum* L. inhibits the body’s production of inflammatory enzymes, which cause pain and other inflammation symptoms [61]. *O. sanctum* L. possesses anti-inflammatory effects comparable to those of ibuprofen, naproxen and aspirin. Prostaglandin synthase turns arachidonic acid into prostaglandins, which causes pain and inflammation. It is generally known that cyclooxygenase (COX) has two distinct isoforms, COX-1 and COX-2, enzymes that convert arachidonic acid to prostaglandins. Furthermore, *O. sanctum* L. decreases uric acid levels in rabbits, reducing gouty arthritis and other joint inflammations [104].

### 4.21. Anti-Ulcer Activity

According to reports, *O. sanctum* L. has significant anti-ulcerogenic and ulcer-healing characteristics [92] that promote mucous secretion while decreasing acid secretion. One may classify *O. sanctum* L. fixed oil as a medication with antiulcer properties that are derived from natural sources. Rats and guinea pigs were used to investigate the anti-ulcerogenic efficacy by Dharmani et al. (Table 7) [92].

### 4.22. Miscellaneous Activities

More than 125 varieties of medicinal plants are utilized in allopathic medications, indicating that medicinal plants are useful not just for the creation of Ayurvedic and Unani medicines but also for allopathic medicines [105]. The anthelmintic activity in an in vitro model utilizing Caenorhabditis elegans is attributed to the essential oil derived from *O. sanctum* L. [106]. *O. sanctum* L. is known to be a good mosquito repellent and to have strong larvicidal properties [79]. *Culex quinquefasciatus*, *Anopheles stephensi* and *Aedes aegypti* are just a few of the mosquito species it is known to be effective against.

## 5. Conclusions

*O. sanctum* L. has a wide spectrum of pharmacological properties, indicating its potency as a therapeutic agent. It has the capacity to alter atherosclerosis-related gene expression while also providing protection against oxidative damage and inflammation due to its powerful antioxidant qualities. The plant has substantial anti-toxic properties, notably against heavy metals such as cadmium, and has promise as a natural anticoagulant similar to conventional medications. Additionally, *O. sanctum* L. has anticancer, antifertility, and immunomodulatory effects, indicating a diverse function in health management. Its effectiveness in treating illnesses like kidney stones and psychological stress highlights its therapeutic adaptability. Overall, *O. sanctum* L. stands up as a remarkable natural therapy with several uses that require additional investigation to fully understand its processes and therapeutic potential.

## Figures and Tables

**Table 1:** Traditional use of *O. sanctum* L. in different disorders.

Uses	Functions/Mechanisms	References
Periodontal disorder	Counteracting oral malodor (halitosis)	[25]
Fever	It destroys protozoa, bacteria and viruses and effectively reduces temperature	[14]
Skin disorder	Prevent ringworm infection	[26]
Respiratory disorder	In cases of bronchitis and asthma, it aids in mobilizing mucus	[14]
Pemphigus	Increasing immunity helps blisters and sores heal faster	[27]
Diabetes	Enhancing insulin secretion and boosting pancreatic beta cell activity	[28]

**Table 2:** Identified bioactive compounds in *O. sanctum* L.

Compound	Structure	Molecular Formula (MW)	Activity	References
Apigenin	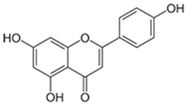	C_15_H_10_O_5_ (270.24)	Stimulate adult neurogenesis	[29]
Carvacrol	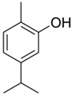	C_10_H_14_O (150.22)	Inhibits the growth of several bacteria strains	[29]
Caryophyllene	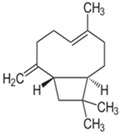	C_15_H_24_ (204.35)	Able to modulate inflammatory processes in humans via the endocannabinoid system	[29]
Cirsimaritin	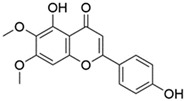	C_17_H_14_O_6_ (314.29)	Cirsimaritin has been shown to inhibit tumor cell growth	[35]
Estragole	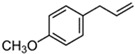	C_10_H_12_O (148.20)	Aromatherapy is widely used and has a strong local anesthetic effect	[29]
Eugenol	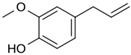	C_10_H_12_O_2_ (164.21)	Eugenol is utilized in fragrances and flavorings. It also serves as a local anesthetic and antiseptic	[36]
Linalool	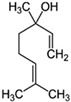	C_10_H_18_O (154.25)	Linalool has anti-inflammatory, anti-nociceptive properties	[29]
Rosmarinic acid	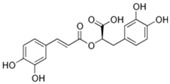	C_18_H_16_O_8_ (360.31)	Because of its capacity to inhibit GABA transaminase, particularly 4-aminobutyrate transaminase, it has the potential to be anxiolytic	[29]
Ursolic acid	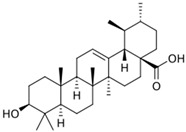	C_30_H_48_O_3_ (456.70)	Ursolic acid shows anti-cancer properties by inhibiting damage repair mediated by vaccinia-related kinase 1 (VRK1) in lung cancer cells	[37]

**Table 3:** A comprehensive overview of the key nutraceutical components of Tulsi and their associated health benefits.

Nutrients/Compounds and Activities	Nutraceutical Value	References
Flavonoids	Potent antioxidant properties; helps reduce oxidative stress and inflammation	[23,41]
Phenolics	Anti-inflammatory and antimicrobial effects; supports immune function	[21,42]
Essential Oils	Adaptogenic properties; aids in stress management	[43]
Antioxidant Activity	Protects cells from oxidative damage; may reduce the risk of chronic diseases	[15,44]
Anti-inflammatory Activity	Helps alleviate inflammation; potential application in managing inflammatory conditions	[42]
Antimicrobial Activity	Exhibits broad-spectrum antimicrobial properties; effective against bacteria, viruses and fungi	[41,42]
Cardiovascular Health	Supports heart health; may help regulate blood pressure and lipid levels	[45,46]
Metabolic Disorders	Potential in managing diabetes and obesity; regulates blood glucose levels and lipid profile	[45,46]
Neuroprotective Effects	Protects against neurological damage; potential application in cognitive health and neurodegenerative diseases	[11,47]

**Table 4:** Preventive measure against malarial activity of *O. sanctum* L.

Part of Plant	Yield of Ethanolic Extract (g)	IC_50_ (µg/mL)	References
Leaf	13.10	35.58	[69]
Stem	2.85	53.50	
Root	1.51	87.40	
Flower	1.64	71.91	

**Table 5:** Antiviral activity of the compounds of *O. sanctum* L.

Compound Name	Susceptible Virus	Virus Type	EC_50_ (mg/L)	Selectivity Index (SI) Value	References
Ursolic acid	Herpes Virus (HSV-1)	DNA Virus	6.6	15.2	[73]
	Adenovirus (ADV-8)	DNA Virus	4.2	23.8	
	Coxsackievirus (CVB1)	RNA Virus	0.4	251.3	
	Enterovirus (EV71)	RNA Virus	0.5	201	
Apigenin	Herpes Virus (HSV-2)	DNA Virus	9.7	6.2	[73]
	Hepatits-B (e) Antigen	DNA Virus	12.8	1.3	
Linalool	Adenovirus (ADV-II)	DNA Virus	16.9	10.5	[73]

**Table 6:** Activity of *O. sanctum* L. against cancer.

Name of Anticarcinogenic Compound	Function	References
Flavonoid vicenin-2 (VCN-2)	Effect on prostate cancer cells that is anti-proliferative, anti-angiogenic and pro-apoptotic.	[76]
Alcoholic extract of the leaves	7,12-dimethylbenzanthracene (DMBA), aflatoxin B1 (AFB1) and Protective action against skin cancer caused by 3 methylcholanthrene (MCA).	[77]

**Table 7:** Anti-ulcer activity of *O. sanctum* L.

Type of Ulcer Induction in Rat	Doses of *O. sanctum* L.	Effectiveness (%)	References
Pyloric ligation (PL)	100 mg/kg of body weight	62.06	[92]
Aspirin (ASP)		63.49	
Alcohol (AL)		53.87	
Cold restraint (CRU)		65.07	
Histamine-induced duodenal (guinea pigs)		61.76	
